# Temperature dependent bacteriophages of a tropical bacterial pathogen

**DOI:** 10.3389/fmicb.2014.00599

**Published:** 2014-11-14

**Authors:** Jinyu Shan, Sunee Korbsrisate, Patoo Withatanung, Natalie Lazar Adler, Martha R. J. Clokie, Edouard E. Galyov

**Affiliations:** ^1^Department of Infection, Immunity and Inflammation, University of LeicesterLeicester, UK; ^2^Department of Immunology, Faculty of Medicine, Siriraj Hospital, Mahidol UniversityBangkok, Thailand

**Keywords:** lysogeny, bacteriophages, *Burkholderia pseudomallei*, *Burkholderia thailandensis*, temperature-dependency

## Abstract

There is an increasing awareness of the multiple ways that bacteriophages (phages) influence bacterial evolution, population dynamics, physiology, and pathogenicity. By studying a novel group of phages infecting a soil borne pathogen, we revealed a paradigm shifting observation that the phages switch their lifestyle according to temperature. We sampled soil from an endemic area of the serious tropical pathogen *Burkholderia pseudomallei*, and established that podoviruses infecting the pathogen are frequently present in soil, and many of them are naturally occurring variants of a common virus type. Experiments on one phage in the related model *B. thailandensis* demonstrated that temperature defines the outcome of phage-bacteria interactions. At higher temperatures (37°C), the phage predominantly goes through a lytic cycle, but at lower temperatures (25°C), the phage remains temperate. This is the first report of a naturally occurring phage that follows a lytic or temperate lifestyle according to temperature. These observations fundamentally alter the accepted views on the abundance, population biology and virulence of *B. pseudomallei*. Furthermore, when taken together with previous studies, our findings suggest that the phenomenon of temperature dependency in phages is widespread. Such phages are likely to have a profound effect on bacterial biology, and on our ability to culture and correctly enumerate viable bacteria.

## INTRODUCTION

It is increasingly accepted that bacterial physiology and evolution are impacted and often driven by phages (Abedon, 2008). Indeed, lytic phages that infect and kill their bacterial hosts shortly after infecting them, are key to shaping bacterial population dynamics by selectively eliminating particular strains of bacteria thus allowing others to dominate (Clokie et al., 2011). Alternatively, the genome of an infecting phage can become incorporated into the chromosome of its host bacterium, or exist as a plasmid (Prentki et al., 1977; Utter et al., 2014) without causing bacterial cell death. In this lysogenic life cycle, the phages can substantially influence bacterial phenotypes (Abedon and LeJeune, 2005). Studies of different phages and their bacterial hosts continue to reveal a remarkable interplay between bacteria and associated phages (Suttle, 2007; Zhao et al., 2013). These detailed interactions between phages and their host bacteria vary significantly, and have only been studied in detail for a few bacterial species of ecological, economic, or medical interest (Abedon, 2008). In this study, we set out to examine the environmental phages that infect the bacterial pathogen *B. pseudomallei*. Before this study, such phages had received little attention. *B. pseudomallei* is the causative agent of melioidosis, a severe and often fatal infection. (Wiersinga et al., 2012).

Although many phages have been isolated from soil, surprisingly little research has investigated the impact of phages on soil microorganisms, despite the ecological, agricultural, and environmental importance of the soil biome. Alongside other diverse organisms, the soil environment is inhabited by several bacterial species that are pathogenic to humans. Such pathogens normally subsist in the soil, but occasionally come into contact with humans and may cause disease (Berg et al., 2005; Baumgardner, 2012). The bacterium *B. pseudomallei* is one such pathogen that inhabits a tropical soil reservoir, where it may be found at densities of up to 10^5^ bacteria per gram of soil. Although *B. pseudomallei* can be found at various depths within the soil profile, the topsoil is considered to be the primary source of infection (Limmathurotsakul et al., 2010; Trung et al., 2011). This pathogen is endemic in many tropical areas; the highest number of cases of human infection is reported in South East Asia and Northern Australia (Limmathurotsakul et al., 2010; Trung et al., 2011).

Determining the factors that control bacterial dynamics in soils is of importance for understanding the disease melioidosis. Previous studies have identified that bacteria in the environment are controlled by physical factors, including temperature, salinity, pH and soil composition, in addition to biotic factors, such as vegetation type (Inglis et al., 2001; Inglis and Sagripanti, 2006; Limmathurotsakul et al., 2013). Little work has been carried out on the phages that infect this organism, and very few phages that are capable of propagating on *B. pseudomallei* have been identified.

Numerous *B. pseudomallei* genomes have been sequenced in order to understand the biology of this pathogen. This has revealed the presence of several temperate phage genomes (Ronning et al., 2010; Kvitko et al., 2012). The aim of this work was to expand on our earlier study (Gatedee et al., 2011), and to focus on the phages that are found in the environment. To do this, we sampled soil from rice paddies from several locations in the North-Eastern part of Thailand where melioidosis is abundant. We isolated several phages that infect *B. pseudomallei* and the related bacteria, *B. thailandensis*. Four of the phage genomes were sequenced, and these are the first described genomes of podoviruses that infect this organism. We provide experimental evidence that the phages have a temperature dependent lifestyle, and that this factor has the potential to impact on the population dynamics and the pathogenicity of *B. pseudomallei*. The discovery of such phages has profound implications for our understanding of bacterial ecology.

## MATERIALS AND METHODS

### ISOLATION AND CHARACTERISATION OF PHAGES

The phages analyzed in this study were isolated from soil samples in Thailand as previously described (Gatedee et al., 2011). In brief, the soil samples were suspended in SM buffer and vigorously mixed. The resulting supernatant was then used for the phage isolation enrichment procedure at 37°C employing *B. pseudomallei* K96243 as a host. From each sampling site, at least 10 samples were collected and assessed for the presence of the phages. Spot assays and plaque assays were then used to isolate the resulting phages, with the latter used to make the phages clonal (Limmathurotsakul et al., 2013).

Stocks of the phages were prepared and analyzed; their morphology was determined by transmission electron microscopy, which was carried out by Stefan Hyman from the Core Biotechnology Services at the University of Leicester. The methodology has been described previously (Shan et al., 2012).

Standard phenol chloroform extraction and isopropanol precipitation techniques were used for phage DNA extraction (Sambrook et al., 1989). Genome sequencing was performed by the Centre for Genomic Research, at the University of Liverpool, UK using Roche 454 technology. Annotation was carried out using Glimmer and GenemarkS to predict coding regions (Delcher et al., 1999; Besemer et al., 2001). The four complete genome sequences, ØBp-AMP1, ØBp-AMP2, ØBp-AMP3, and ØBp-AMP4, were deposited in the EMBL database under the Accession Numbers of HG793121, HG796219, HG796220, and HG796221, respectively. The major capsid proteins were identified bioinformatically using BLASTp searches in the NCBI database (Tamura et al., 2007; Kumar et al., 2008). The most similar sequences to phage ØBp-AMP1 were then analyzed in MEGA (Molecular Evolutionary Genetics Analysis) version 5.2.2. Results from the neighbor joining analysis are presented, with a bootstrap analysis of 1,000 replicates. The phylogenetic trees were rooted with bacteriophage T7 as an outgroup. Maximum likelihood and parsimony analysis were also carried out. Phages were compared to each other at the nt level using the Artemis Comparison Tool (ACT; Carver et al., 2005).

### STUDIES OF BACTERIA-PHAGE INTERACTIONS

To assess phage impact on bacterial growth at different temperatures, three biological replicates of *B. thailandensis* E264 cultures were grown at 37°C to mid-log phase and each was split into four aliquots. Two of each of the aliquots were then infected with ØBp-AMP1, and two non-infected aliquots were used as a control. For each replicate, an infected and a non-infected control culture were incubated at 25°C, and a second set at 37°C for 6 h. The cultures were sampled hourly to estimate the bacterial density by measuring the OD_600_. The corresponding phage counts in the media were enumerated by counting the number of plaque forming units (PFU).

To determine how phage infection affects the ability of *B. thailandensis* to form colonies on a solid media, *B. thailandensis* E264 was grown to mid-log phase in liquid media and split into two aliquots. One aliquot was infected with ØBp-AMP1 at an MOI of 10 for 10 min, and serially diluted samples were plated onto two sets of LB agar plates. One set was then incubated at 25°C and the other at 37°C. Non-infected cultures were used as a control.

To assess stable lysogens, *B. thailandensis* E264 was infected with the phage, and the bacteria were plated on LB agar and incubated at 25°C. A colony was determined to be phage positive using PCR. The bacteria were then propagated at 25°C and assessed in both *in vitro* and *in vivo* experiments. For the *in vitro* experiments, the lysogenic bacteria were grown in liquid medium at 25°C to mid-log phase. For each of the two identical plates, 1 ml of the culture medium (approximately 10^6^ bacteria) was mixed with 8 ml of melted 0.4% (w/v) agar precooled to 45°C, and distributed evenly to solidify on the agar surface of a base plate. One of the resulting plates was then incubated over night at 25°C, and the other at 37°C. The phage binding efficiency at 25°C and 37°C was determined according to a previously published method (Jia et al., 2010). In order to test if phages can still inject DNA into the bacteria at 25°C, ferrous ammonium sulphate (FAS) was used as a virucide to kill free phages, and phages inside the cell were tested using plaque assays (McNerney et al., 1998).

The two sets of PCR primers used in this study were designed using Primer-Blast. One targets the *mazG* gene (F_mazG210_: CACACAGGCAGCAGTCAAGT; R_mazG210_: GCGTACTTCCTCCGATACGA), the other one targets the phage *ttpB* gene (Gatedee et al., 2011). Both primer sets are specific to the phages identified in this study. PCRs were carried out in a LabCycler (SensoQuest GmbH, Göttingen, Germany) in total volumes of 50 μl, containing 0.25 mM dNTPs, 3 mM MgCl_2_, 2 μM primers, 50 ng of template DNA, 0.5 unit of Taq polymerase (Bioline, UK), and 5 μl 10 × Taq buffer (Bioline, UK). Amplification conditions were: 94°C for 2 min, 35 cycles of 94°C for 45 s, 51°C for 45 s, and 72°C for 1 min, followed by a final extension of 10 min at 72°C. PCR products were analyzed on a 1% (w/v) agarose gel.

For the animal experiments, a phage positive colony maintained on LB agar at 25°C was suspended in physiological saline, and each of the five mice (C57Bl/6) were inoculated intravenously via the latteral tail vein with ∼10^6^ bacteria in 100 μl. The mice were sacrificed at 24 h post infection. The harvested spleen was homogenized in 2 ml PBS in a Stomacher 80 Biomaster (Steward Ltd, UK) using 2 cycles of 30 s. 200 μl of the neat homogenate was spread plated, and the plates were incubated at 25°C. The resulting colonies (15–20 for each sample) were assessed for the presence of the phage by PCR using the primers described above.

All investigations involving animals were carried out according to the requirements of the Animal (Scientific Procedures) Act 1986, including obtaining ethical approval from our local (University of Leicester) ethical review process. Mice were monitored for signs of disease during the experiment and humanely culled when pre-defined end-points were reached by cervical dislocation according to Schedule 1 of the Animal (Scientific Procedures) Act 1986.

## RESULTS

### ISOLATION AND CHARACTERISATION OF PHAGES

**FIGURE 1:**
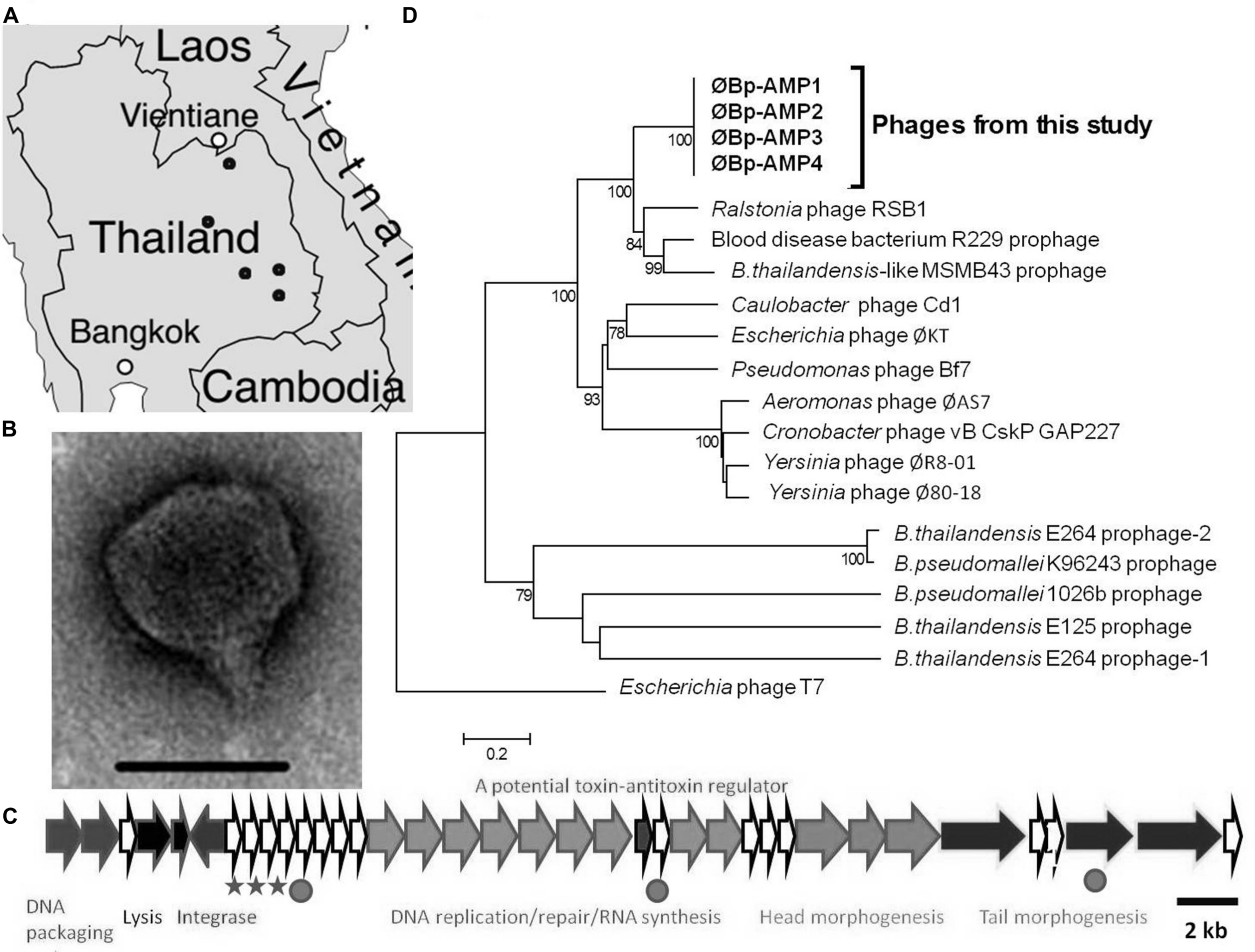
**Novel *B. pseudomallei* and *B. thailandensis* podoviruses are abundant in the pathogen endemic area of Thailand. (A)** Numerous phages were isolated from soil samples (∼sampling locations are highlighted by dots). **(B)** TEM of a podovirus, typical of phages isolated in this study. Scale bar is 50 nm. **(C)** Whole genome sequencing of four phages revealed an almost identical genome arrangements and content as illustrated here for ØBp-AMP1, the SNPs are shown with stars and the Indels with circles. Genomes have been deposited in EMBL. **(D)** Phylogenetic analysis based on the capsid protein for the four fully sequenced phages showing that the phages are novel and closely related to phages. Scale bar represents 0.2 amino acid changes per site.

To study *B. pseudomallei* phages present in the environment, we sampled soil from rice paddies throughout North-Eastern Thailand and isolated numerous phages capable of infecting this bacterium (**Figure 1A**). Transmission electron microscopy revealed that most of the isolated viruses were podoviruses (**Figure 1B**). All of these podoviruses were morphologically similar to the phage that we previously described (Gatedee et al., 2011). Each of these phages could infect the *B. pseudomallei* K96243 strain and the related non-pathogenic *B. thailandensis* strain E264, although the efficiencies by which they infected the two species varied (data not shown).

Four of the podovirus genomes were selected to be sequenced on the basis of representing a wide geographical distribution as well as having differences in their plaquing efficiencies on the two bacterial species. The data revealed that the phages form a novel group of highly related viruses (**Figures 1C,D**). The genome architectures are conserved as shown in **Figure 1C**. When the genomes were compared using the ACT, they were found to be almost identical and only varied by three single nucleotide polymorphisms (SNPs) and three short insertions/deletions (Indels; **Figure 1C**). All SNPs and one Indel are located in the hypothetical proteins between the predicted integrase ORF and the DNA replication/repair module (**Figure 1C**). The second Indel is found within the DNA replication/repair module, specifically, it was in a hypothetical protein located between a DNA polymerase and an exonuclease. The final Indel was found within an ORF coding for a putative transglycosylase. Among the three SNPs, two would translate as a non-synonymous mutation and one a synonymous mutation in the resulting amino acid.

The phages encoded a tyrosine recombinase/integrase, which suggests that they can access the lysogenic lifestyle. Phylogenetic analysis was carried out on the capsid genes using neighbor joining and Maximum Likelihood analysis. Both analyses gave very similar tree topology and the results from the neighbor joining are presented. Despite this observation and the apparent abundance of these phages in the environment, no publicly available genomes of *B. pseudomallei* or *B. thailandensis* carry such phages (of the genomes of 46 *B. pseudomallei* strains and 6 *B. thailandensis* strains, present in NCBI, August 2014).

### STUDIES OF BACTERIA-PHAGE INTERACTIONS

To interpret why this phage group was not previously observed, we assessed phage-host interactions under different experimental conditions using *B. thailandensis*, a recognized model organism for *B. pseudomallei* (Haraga et al., 2008). The studies reported here focus on the impact of temperature on these interactions as this is a known variable affecting the bacterium in the environment. *B. pseudomallei* encounters different temperatures in the soil environment due to daily and seasonal variations, and it is also encounters the more consistent higher temperature when infecting warm-blooded hosts. To determine if the infection parameters of podoviruses differed with temperature, we conducted a detailed set of phage-bacterium interaction studies at 25°C and 37°C. These temperatures are representative of the colder and warmer end of the temperature range at which the phage may encounter *B. pseudomallei* in the natural environment. Furthermore, 37°C has been shown to be the optimal temperature for propagation of the majority of *B. pseudomallei* strains under laboratory conditions (Chen et al., 2003).

**FIGURE 2:**
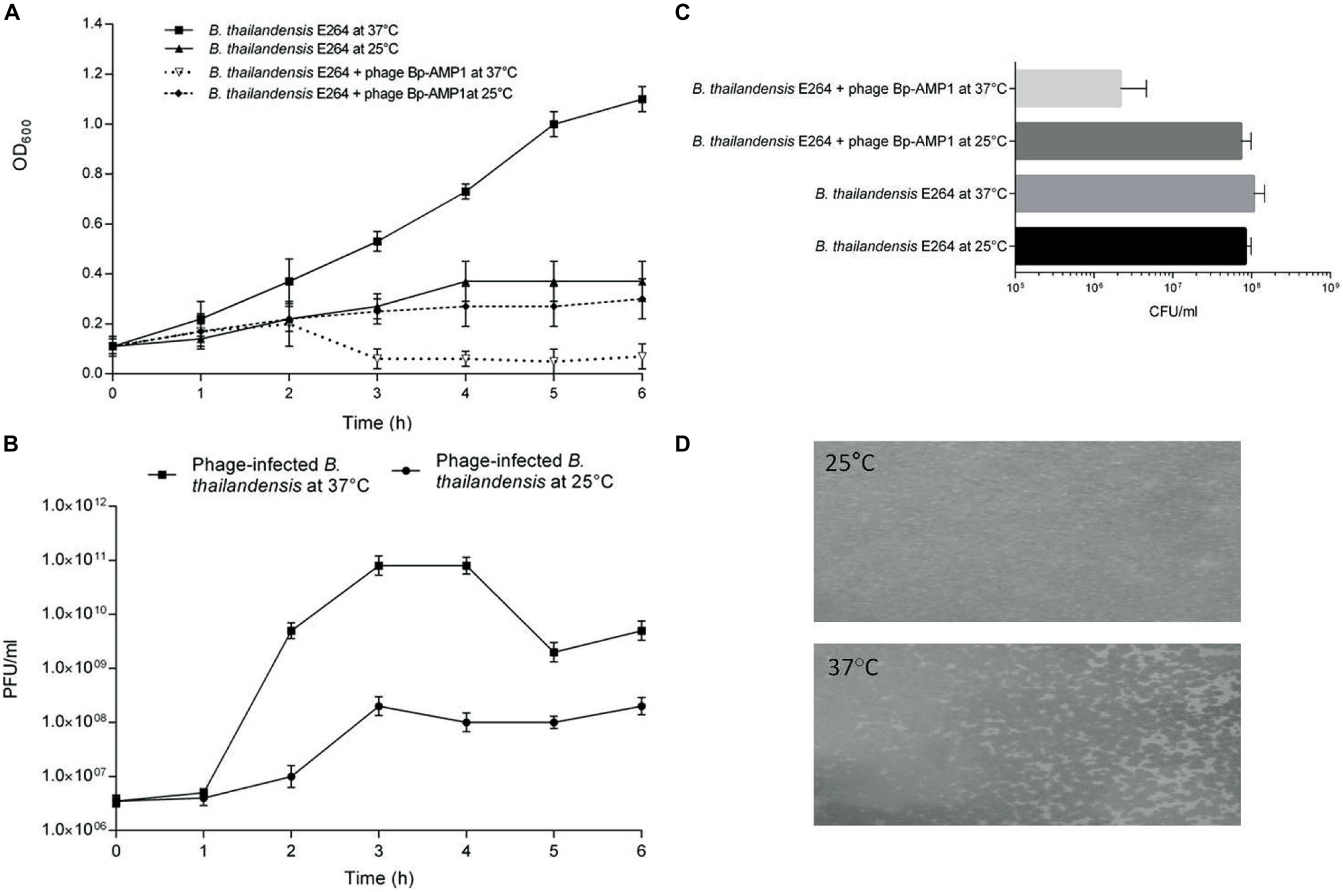
**Temperature-dependent lysogeny of *B. thailandensis* by ØBp-AMP1. (A)** *B. thailandensis* E264 was grown to mid-log phase and split into four aliquots, two were then infected with ØBp-AMP1, and two non-infected cultures were used as a control. The cultures were incubated either at 25 or 37°C for 6 h and sampled hourly to estimate the bacterial density by measuring the OD at 600 nm. **(B)** Corresponding phage counts in the media were enumerated by counting the number of PFU. Data revealed that at 37°C, the phage lysed the host bacteria and produced phage progeny at titres of ∼10^11^ PFU/ml after 1 h of infection whereas at 25°C, most of the bacteria were not lysed and the infected bacteria produced phages at the significantly lower titre of ∼10^8^ PFU/ml. Results for each data point are the mean of three biological replicates each performed in triplicate ±SD. **(C)** *B. thailandensis* E264 was grown to mid-log phase in liquid media and split into two aliquots. One aliquot was infected with ØBp-AMP1 at an MOI of 10 for 10 min and spread in serial dilutions onto two sets of LB agar plates. One set was then incubated at 25°C and the other at 37°C. Non-infected cultures were used as a control. At 25°C, infected and non-infected cultures yielded essentially the same number of colonies. However, at 37°C the mean of the infected culture was 2.17 × 10^6^ (SD 2.47 × 10^6^) CFU/ml compared to 7.33 × 10^7^ (SD 2.52 × 10^7^) for the infected culture at 25°C, 1.07 × 10^8^ (SD 4.04 × 10^7^) for the uninfected culture at 37°C and 8.33 × 10^7^ (SD 1.53 × 10^7^) uninfected culture at 25°C. Results plotted are these means from three biological replicates ±SD. **(D)** Stable lysogens of *B. thailandensis* E264 were selected and maintained on agar plates incubated at 25°C. The lysogen culture was then grown in liquid media at 25°C to mid-log phase. Approximately 10^6^ bacteria were then mixed with 8 ml of melted 0.4% (w/v) agar precooled to 45°C, and plates were cast and incubated at either 25 or 37°C. A thick bacterial lawn was observed at 25°C, but substantial phage mediated lysis was observed at 37°C. Representative images from at least three independent experiments are shown.

To probe the impact of temperature on phage-host dynamics, liquid bacterial cultures were infected with phage ØBp-AMP1 (Gatedee et al., 2011) and incubated at either 25°C or 37°C with non-infected cultures used as a control. At 37°C, the phage caused rapid bacterial lysis, whereas when incubated at 25°C, both the phage-infected and non-infected bacteria grew steadily (**Figures 2A,B**). Adsorption assays showed that there were no difference in terms of binding efficiency as ∼90% of all of the phages bound to bacterial cells within 45 min at both a temperature of 25°C and at 37°C. Furthermore, experiments using the virucide FAS confirmed that the phage can still inject DNA into host cells at both temperatures. This suggests that the phage infection cycle is temperature dependent, with a higher temperature permissive for lytic infection and the lower temperature for lysogenic infection.

To further assess the impact of temperature on the outcome of phage infection, plate assays were utilized. To do this, *B. thailandensis* cultures were infected with ØBp-AMP1 in liquid media for 10 min at 37°C, and serially diluted samples were plated and incubated at 25 or 37°C. The phage-infected and control samples that were incubated at 25°C, and the non-infected control incubated at 37°C, yielded essentially the same number of colonies (**Figure 2C**). In contrast, the phage-infected sample incubated at 37°C yielded ∼2 logs fewer colonies, this difference is statistically significant with a P-value of <0.01 (**Figure 2C**). Furthermore, of the 50 selected colonies that originated from the infected sample that grew at 25°C, 40 were positive for phage presence as assessed by PCR with phage specific primers.

The stability of these lysogens was then characterized both *in vitro* and *in vivo*. In both cases the lysogen colonies were sub-cultured three times at 25°C, and confirmed as phage positive using PCR. When these lysogens were grown in liquid media, ØBp-AMP1 was detected in the culture supernatant at 37°C but not at 25°C. Furthermore, when a lysogenic bacterial stock was embedded in 0.4% (w/v) agar and incubated at 25°C, confluent bacterial growth resulted. However, at 37°C, only a patchy bacterial growth was observed, which is indicative of the lysogens being induced into the lytic cycle (**Figure 2D**).

Finally, to investigate the lysogen behavior *in vivo*, *B. thailandensis* bacteria from a PCR positive colony were suspended in physiological saline, and five mice (C57Bl/6) were inoculated intravenously with ∼10^6^ bacteria. *B. thailandensis* is a good model for *B. pseudomallei* because the two bacterial species are genetically closely related, and although *B. thailandensis* is not capable of causing disease in humans, it can cause disease signs in mice (Deshazer, 2007; Haraga et al., 2008). The mice were sacrificed at 24 h post infection, and homogenized spleen samples were plated and incubated at 25°C. The resulting colonies (15–20 for each sample) were assessed for phage presence using PCR. No positive colonies were detected from samples from 4 of the mice, and only 3 of 20 colonies were phage-positive in the fifth sample. This demonstrated an *in vivo* selection against bacteria containing phages. It seems likely that only bacteria that lose their phage are capable of maintaining infection in a warm blooded animal.

## DISCUSSION

Before our previous work (Gatedee et al., 2011) and this report, the only phages known to infect *B. pseudomallei* were all myoviruses (Yordpratum et al., 2011). In contrast, we isolated numerous podoviruses and demonstrated that they are common in tropical soil where they infect *B. pseudomallei* and *B. thailandensis*. We have shown that many of the phages are variants of a commonly occurring podovirus, and that they have a temperature dependent lifecycle at two contrasting environmentally relevant temperatures. It is highly plausible that these phages therefore have a profound and hitherto unrecognized effect on the population dynamics of bacteria. There are daily and seasonal periodic variations in temperature from 20°C to 37°C and above in the top-soil temperature in wet rice fields, a natural habitat for *B. pseudomallei*. It has been suggested that the optimal temperature for growth of the pathogen is 37–42°C; by implication at such temperatures the highest numbers of bacteria will be present. It is therefore likely that the phage studied in our work has evolved to switch from a lysogenic to a lytic life style when the host bacteria are abundant. The induction of the phage during the bacterial infection of warm-blooded hosts may be caused as a result of temperature shift and the stress experienced by the bacteria as they enter this environment.

The observation that temperature can influence the outcome of phage-bacterial interactions has previously been made for other bacteria-phage systems. For example, temperature dependent restriction modification systems in industrially important lactobacilli are only active at lower temperatures, rendering bacteria less sensitive to phage infection (Sanders and Klaenhammer, 1984; O’Driscoll et al., 2004), and more lysogens seem to be present at low temperatures in aquatic cyanobacteria than there are at higher temperatures (McDaniel et al., 2006; Chu et al., 2011). Also of relevance to this work are studies on temperature sensitive mutant derivatives of lambda phage, which have also shown that a temperature shift causes such phages to be induced into the lytic cycle (Groman and Suzuki, 1962; Eshima et al., 1972; Lieb, 1979). To the best of our knowledge, our observation is the first time that a clear shift for naturally occurring phages to enter a lysogenic or a lytic lifestyle depending on the temperature at which they coincide with their hosts has been demonstrated. Our work builds on previous descriptions of the importance of temperature to host-phage dynamics (Groman and Suzuki, 1962; Eshima et al., 1972; Lieb, 1979; Suzuki et al., 2002; McDaniel et al., 2006; Chu et al., 2011) and suggests that temperature dependent phage infections are widespread in nature. Thus, the scale and impact of the phenomenon of temperature dependent phages is likely to be very significant.

The findings presented here have several major implications for *B. pseudomallei*. The first relates to the environmental monitoring of the pathogen in which most methods of bacterial enumeration rely on selective growing of these bacteria at 37–42°C (Limmathurotsakul et al., 2013). Our data suggest that lysogens present in the natural bacterial population are unlikely to survive this isolation process due to phage activation; thus, there could be a major underestimation of bacterial numbers. This underestimation may also be further compounded by these phages, which once induced and released from their host bacteria, could subsequently infect any phage-free bacteria within the samples. Thus, the impact of these phages could cause a significant underreporting of the estimate of *B. pseudomallei* in the environment.

Our experiments also imply that phage-free bacteria are relatively more infective to human hosts than lysogens are. This is due to these temperate phages undergoing induction into the lytic cycle upon entry into a warm-blooded host. Therefore the relative infectivity of the inoculum if lysogens are present would be significantly lowered. This also would explain the observation that only the bacteria that are free from these podoviruses have been isolated and cultured from clinical samples.

The ability for this group of phages to lyse their host cells at higher temperatures is consistent with the apparent absence of lysogens in the strain collections, and also explains the lack of such phages in *B. pseudomallei/B. thailandensis* genome sequences. Thus, despite a substantial genome sequencing effort, this key aspect of bacterial diversity has not been revealed. The phenomenon of temperature dependent lysogeny could be an important element of the biology and pathogenesis for many other bacterial species, including other pathogens that can inhabit an environmental reservoir. Indeed the ‘great plate count anomaly’ (Staley and Konopka, 1985), that states that we can currently only culture 0.5–1% of all bacteria found in the natural world, could at least in part be explained by the impact of conditionally dependent phages.

## Conflict of Interest Statement

The authors declare that the research was conducted in the absence of any commercial or financial relationships that could be construed as a potential conflict of interest.
